# ESG activity recognition enhances organizational commitment and service-oriented organizational citizenship behavior among insurance call center staff

**DOI:** 10.1016/j.heliyon.2024.e31999

**Published:** 2024-05-31

**Authors:** Serin Choi, Kyeong-Sook Jeong, So Ra Park

**Affiliations:** a Cheju Halla University, 38 Halla University Rd., GumhoSaeGye Education Building A-408, Jeju-si, Jeju-do, South Korea, 63092; b Chonnam National University, 77 Yongbong-ro 1st Business Building, 4th BK21 Education & Research Group of Subtraction Platform Office, Buk-gu, Gwangju, South Korea, 61186; c Chonnam National University, 77 Yongbong-ro, 2nd Business Building Office #412, Buk-gu, Gwangju, South Korea, 61186

**Keywords:** ESG activity recognition, Service-oriented organizational citizenship behavior (SO OCB), Organizational commitment (OC), Self-efficacy, Empowerment, Ethical climate theory

## Abstract

Service-oriented organizational citizenship behavior refers to service workers' helping, cooperating, sharing, and donating actions that benefit others at a cost to themselves. Based on ethical climate theory, this research investigates whether corporations adopting environmental, social, and governance (ESG) management enhance service-oriented organizational citizenship behavior (SO OCB) among service employees. A total of 230 surveys were collected from call center workers in the insurance industry, and STATA 14.0 was used to analyze the 204 responses with useable data. The results show that employees' recognized ESG activities enable SO OCB through organizational commitment. Additionally, ESG activity recognition has a positive relationship with self-efficacy and empowerment, which helps service employees regulate external expectations. Thus, this finding is significant for call center workers experiencing emotional labor. Furthermore, the results suggest that firms can contribute to employees' SO OCB by practicing ESG activities. Firms should inform employees of their ESG management efforts as employees' recognition of an ethical climate can enhance their willingness to perform service-oriented behavior. Finally, ESG activity recognition can increase employees’ organizational commitment, an important predictor of employee satisfaction and negative turnover rates.

## 1 Introduction

Organizations are under pressure to adopt ESG (environment, social, and governance) practices to adhere to regulatory requirements, attract financial investment, realize expected positive outcomes, and respond to the desires of shareholders, customers, and other stakeholders. ESG management involves substantial resources and intense organizational change. Many studies have suggested that positive financial performance is an outcome of ESG [1]. However, measuring investment-related improvements as ESG outcomes can be challenging. In addition, non-financial outcomes of ESG management lack unified measurements, and performance is hard to evaluate objectively [2].

In support of producing evidence of non-quantifiable ESG performance areas, scholars are beginning to view employees' perceptions of corporate ESG practices and recognition of the related corporate activities as proxies of ESG/corporate social responsibility (CSR) management [[3], [4], [5]]. Stakeholders' perception and recognition of corporate ESG management refers to how they view and interpret a new organizational culture after adopting ESG. Organizations' green management practices can enhance employees' perceptions regarding the organizational climate, resulting in in-role and extra-role green behaviors [6]. A psychological work climate represents employees’ perceptions of their work environment [7]. The current research uses ESG activity recognition as a proxy for ESG-driven organizational climate and examines its effects on employee motivation, organizational commitment (OC), and service-oriented organizational citizenship behavior (SO OCB).

This study's motivation is based on the following academic contributions. First, it aims to investigate the largely ignored area of internal stakeholders' attitudes and behaviors, whose interests are at the heart of the ESG movement. Second, studying employees' ESG perception and recognition provides the ability to link ESG management with performance in non-financial areas. A company's CSR is generally recognized as corporate prosocial efforts in support of relevant stakeholders, including employees and customers [8]. This research attempts to identify whether enterprise-wide acceptance of ESG management can influence employee's recognized organizational climate and their attitudes toward resources, other stakeholders, and governance structures.

Furthermore, this research aims to contribute to relieving employees' emotional labor. It recognizes an organization's ESG activities as inducing self-efficacy and empowerment, motivational constructs that remedy emotional labor among service workers [[9], [10], [11]]. Self-efficacy and empowerment are essential for employees to enhance feelings of control and power, inhibiting emotional labor and promoting these traits [11]. Service worker turnover is a serious human resource issue, and OC is an important predictor of employee turnover and satisfaction [5]. Finally, this research provides organizations with practical approaches for motivating service employees, reducing emotional labor, enhancing OC and its positive outcomes, and garnering SO OCB. Service worker turnover is a serious human resource issue, and OC is an important predictor of employee turnover and satisfaction [1].

To investigate the influence of ESG management adoption on OC and SO OCB, this study focuses on the insurance industry in South Korea. Customer representatives in the insurance industry are knowledge professionals whose jobs are characterized by continuously changing tasks and emotional labor. They are the contact points between companies and their customers. To compensate for the low efficiency and productivity of call centers [12], the insurance industry has adopted AI advisor systems [13], work management systems (including employee monitoring systems), insurance analytics, blockchain technology, and chatbots [14]. Although customer representatives play critical roles for companies, those working in the insurance industry experience workplace challenges such as adopting fast-changing technologies, the threat of job replacement with AI chatbots, invasive work monitoring technology, and customer incivilities causing emotional exhaustion and low job performance [15].

This study aims to determine how a workplace adopting ESG practices encourages OC and SO OCB among high-tech call center customer representatives. Specifically, the current study investigates whether employees' recognition of ESG activities, a proxy for an ESG-changed organizational climate, 1) enhances self-efficacy and empowerment, two motivational factors contributing to OC, and 2) builds OC. Next, the research tests whether 3) employees' SO OCB is a result of 1) and 2). Finally, the research examines 4) whether OC mediates the relationship between employees’ recognition of ESG activities and SO OCB. While not tested in this study, an ESG-driven organizational climate is expected to reduce emotional labor when self-efficacy and empowerment are strengthened.

## 2 Literature review

### 2.1 ESG activity recognition and service-oriented organizational citizenship behavior (SO OCB)

To encourage socially responsible management practices, efforts to promote the incorporation of ESG began in 2005 by the UN Secretary-General at the time, Kofi Anan, and leaders of major financial institutions, who together created the UN's Principles for Responsible Investment (PRI). As of 2021, 3826 financial institutions had joined, promising to consider ESG issues in investment analysis and decision-making.

ESG practices change the ethical climate of a company. According to ethical climate theory, ethical climate is “a type of work climate that is best understood as a group of prescriptive climates reflecting the organizational procedures, policies, and practices with moral consequences” [16]. ESG adoption and management change organizational decision-making and practices, and ethical concerns regarding ESG are often related to an organization's ethical climate and leadership [17]. Adopting ESG management changes the decision-making priorities to minimize the negative impacts on diverse stakeholders. These changes in value can shift the overall culture. Sustainable transformation within an organization requires changes in governance, which involve disruptive innovation and systemic shifts [18]. Systemic change includes infrastructure, institutions, products, markets, social practices, and cultural change.

Ethical climate theory discusses how an individual's behavior is influenced by environmental stimuli [19]. It shows that what employees perceive as correct behavior shapes their moral criteria regarding ethical issues [20]. An ethical climate is defined as employees' shared perceptions of their firms' ethical practices, policies, and procedures [16]. It directly impacts affective states, OC, and job satisfaction and can also influence psychological well-being and dysfunctional behavior [16]. Individuals' normative guidelines show their collective self-concept, related organizational membership, and OC [21]. An ethical culture can predict employees' OCB [22] and in-role and extra-role behaviors [23]. OCB is shaped by employees' perceptions of their organizational culture. An employee's perception of a positive ethical climate helps build psychological bonds with co-workers and the organization through effective identification [22]. Cullen et al. [20] stated that the best way to measure an organization's ethical climate is by asking its employees. Therefore, the optimal method to learn about an ethical climate derived from an organization's ESG adoption is through employees' perceptions.

Hur et al. [24] used higher-order superordinate employee CSR perception regarding customers, shareholders, supervising boards, employees, and society. Ko et al. [4] viewed employees' CSR perception as “the positive perceptions of an organization's identity due to its CSR activities.” They investigated hotel service employees' views of CSR and its role in sequentially increasing organizational identity and OCB. They considered overall perceptions of external CSR management, such as whether a hotel is socially responsible, concerned with improving society, and environmentally responsible. Jin and Kim [3] surveyed employees of Korean manufacturing companies. They defined ESG recognition as “the organizational employees' recognition state to practice a business that can thrive within society with transparent governance.” They investigated how employees' recognition of corporate ESG activities positively influenced their job performance directly and indirectly through change support behavior and innovative organizational culture.

Employees have the ability and means to influence ESG policies and extend its benefits to other stakeholders [25]. Thus, employees are key stakeholders in CSR [26] and are one of the driving forces behind corporations' socially responsible actions [27]. Further, organizations practicing better ESG may be perceived as more attractive to potential and incumbent employees [28]. Understanding how ESG changes an organization's ethical climate and how employees' attitudes and behavior are affected by an ESG-driven ethical culture is important for all organizations.

### 2.2 The regulation of emotional labor through self-efficacy and empowerment

Employees experience less emotional labor when there is an absence of dissonance. Emotional labor is “the process of regulating both feelings and expressions for organizational goals” [29]. Dissonance is a state of disagreement between external job demands and personal feelings that causes psychological discomfort. Employees experiencing dissonance seek consonance [30] by internalizing the emotions mandated by organizations or customers [31]. The process of finding consonance is a burden; hence, the term ‘emotional labor’ was coined. Emotional labor's relationship with OC has been determined to result in less emotive dissonance [32].

In frontline service jobs, emotional labor results from organizations’ control over workers' interactions with customers and a lack of autonomy and balance. Power is a belief in personal self-efficacy and an intrinsic need for self-determination [33]. Regaining a sense of self and a power balance is important for emotionally-labored workers to have positive work experiences and be motivated to continue their work satisfactorily [11]. In this context, motivation can inhibit the formation of emotional labor by empowering employees and boosting their self-efficacy.

Self-determination theory shows how individuals are motivated to engage in activities and explains how employees' autonomy relates to positive job outcomes, satisfaction, emotional exhaustion, and turnover intentions [34]. Intrinsically motivated people engage in behaviors for the pleasure of performing an activity [35]. According to Grandey and Melloy [9], job autonomy is an organizational factor that negatively influences emotional labor. Employees with higher autonomy may form self-determined reasons for introjected jobs and demonstrate better performance [36]. Empowerment encompasses employees’ autonomy in decision-making regarding duties and tasks [37]. Therefore, although they may not value the imposed roles or tasks personally, empowered individuals can easily internalize organizational values and engage in tasks without feeling coerced.

Controlling events creates an external locus of causality, promotes perceived incompetence, and deters intrinsic motivation [35]. Employees working for extrinsic and introjected reasons may perceive their behavior as coerced [38]. According to social cognitive theory, self-efficacy represents the skills one can employ to handle job-related tasks and challenges [39,40]. Pugh et al. [10] found that self-efficacy builds self-confidence in frontline workers and is essential for performing surface acting without adverse effects. Self-control promotes and positively correlates with self-efficacy [41]. Thus, self-efficacy is a desired motivational construct demonstrating one's ability to perform organizational tasks.

Additionally, social cognitive theory discusses how controlled motivation handles the internalization process using self-efficacy. According to Shahriari et al. [42], organizational culture changes can result in empowerment. Hence, organizational changes like ESG management can cause structural shifts that empower employees. Psychological empowerment, in turn, results in diverse performance outcomes. Thus, empowerment and self-efficacy are necessary and important motivational traits for service employees in regulating organizational goals to reduce cognitive resonance within an ESG culture.

### 2.3 Organizational commitment (OC)

OC is vital for predicting positive employee behaviors that contribute to organizational success. It is defined as “the relative strength of an individual's identification with and involvement in a particular organization” [43]. OC is linked with organizational performance outcomes [44], including employee turnover [5,45], engagement [46], performance [47], and job satisfaction [48]. Individual identification is the process of learning about oneself by seeking answers to questions such as how to relate to others and find meaning. It involves aligning one's self-concepts with the organization's [49]. Individuals form OC through the congruence of goals between leaders and followers, which can reduce their intention to leave an organization [45]. OC directly enhances job satisfaction and indirectly impacts employee performance through satisfaction [48].

In addition to its many employee and organizational outcomes, OC has a positive relationship with OCB [50]. In the hospitality context, hotel employees' perception of CSR results in organizational identification, influencing their OCB [4]. According to Cropanzano and Mitchell [51], when employees perceive support from their organization, they tend to exchange it for OC. Further, perceived organizational support closely relates to and predicts OC. Similarly, it was found to influence OC and, subsequently, SO OCB among employees in Taiwanese call centers [52]. Further, Sumardjo et al. [53] found that OC mediates the influence of organizational support and culture on 10.13039/100012782OCB. Therefore, constructs contributing to positive organizational cultures, such as perceived organizational support and employees' recognition of an organization's behavior, are predictors of OC.

OC is essential in explaining how HR practices influence employee outcomes, a major black box problem in management research [46]. Furthermore, according to a meta-analysis study on OC by Mehra [54], it is a useful dependent variable affected by emotional labor. Therefore, OC is an optimal dependent variable for testing employees’ recognition of ESG management.

## 3 Hypothesis development

### 3.1 The operationalization of ESG-driven organizational culture as ESG activity recognition

This research operationalizes ESG-driven organizational culture with employees' ESG activity recognition. The E (Environment), S (Social), and G (Governance) of ESG represent organizations' attempts to enhance sustainability by identifying the means to ensure stakeholders' values and providing a governance structure to implement it. ESG activity recognition refers to “the level to which the employees recognise corporate ESG strategy and commitment” [3]. A higher degree of ESG activity recognition is achieved when an organization makes efforts to ensure employees value its related goals. Therefore, employees' recognition of an organization's increased ESG activities helps dictate corporate culture, as they feel the organization values the environment and the relevant stakeholders' needs and has a sound corporate governing mechanism. Thus, employees' recognition of ESG activities should reflect the organization's dedication to ESG concerns and an ESG-driven organizational culture.

### 3.2 Proposed hypotheses

Self-efficacy is the perceived level of trust an individual has in their ability to complete given tasks [55]. A recent study on Pakistan's public sector banks found that corporate culture factors influence self-efficacy and, in turn, OC [56]. Similarly, organizational culture positively influences self-efficacy and thus positively affects restaurant industry employees' OCB [55]. Regarding customer contact staff, ESG activities can enhance employees' sense of control and confidence, thereby increasing their self-efficacy [55]. Therefore, H1 is proposed as follows.

H1 ESG activity recognition positively relates to employee self-efficacy.

Psychological empowerment is defined as “a subjective, cognitive, and attitudinal process that helps individuals feel effective, competent and authorized to carry out a task” [57]. 10.13039/100014337Furthermore, multiple studies have shown that social and organizational support empowers employees [57]. Empowered employees have the power originally possessed by management [33]. According to Chen et al. [58], to prevent any catastrophic ethical disaster within an organization, ethical expectations should be structured as a system, and individual employees should be empowered to practice ethical behavior according to a given standard. Therefore, for an organization's ESG activities to be recognized by employees, significant efforts must be made to implement ESG as an ethical system. Following this, employees must be empowered to practice the associated activities themselves. Sigler et al. [59] tested whether two dimensions of positive organizational culture influenced workers' empowerment in the US textile industry. The results indicated that organizational culture significantly and positively influences empowerment. Thus, an organization with good ESG standards will empower employees. Therefore, our research proposes H2 as follows.

H2 ESG activity recognition positively relates to employee empowerment.

A high-performing organizational culture can result in employee satisfaction, motivation, and, subsequently, OC [47]. According to Hijal-Moghrabi et al. [60], developing an ethical work culture is essential for promoting high performance within an organization with strong financial and non-financial results. Empowerment and self-efficacy are motivational constructs [33,61] that can be influenced by corporate ESG activities. OC is an attitudinal reaction to psychological empowerment [57]. Empowerment can cause employees to selflessly perform their job responsibilities [62]. It can also increase OC and performance for textile industry employees [59]. Recognized corporate ESG activities can positively influence empowerment, self-efficacy, and organizational immersion indirectly. Therefore, we propose H3 and H4 as follows.

H3 Self-efficacy positively relates to OC.

H4 Empowerment positively relates to OC.

A positive organizational culture can result in OC among employees. Jehanzeb and Mohanty's [63] research demonstrated how organizational justice and employees' perceptions of fairness influence OCB via OC. Furthermore, Oh et al.’s [5] research on hotel employees shows that ESG perception can lead to OC via intrinsic motivation. Moreover, a green cultural shift can promote OC directly and indirectly through employee satisfaction [42]. A study on Ecuadorian workers showed that a firm's economic, social, and environmental CSR builds employees' trust and intrinsic motivation, increasing OC [64]. Regarding overall CSR perception, Ko et al. [4] saw how global CSR perceptions lead to developing organizational identity and OCB sequentially. They found that gender, age, and work experience have moderating influences. They also determined that the relationship between CSR perceptions and organizational identity is stronger for older female employees with more job experience. Youn et al. [65] demonstrated that CSR perception directly and positively influences OC. Similar to Youn et al.’s [65] research, we believe ESG activities recognized by employees working in customer contact services will demonstrate higher OC levels. Therefore, we propose H5 as follows.

H5 ESG activity recognition positively relates to OC.

Employees who internalize organizational goals through OC should demonstrate OCB towards other stakeholders, such as customers [63]. According to Paruzel et al. [66], employees within a company with high CSR recognition increase their OCB only through organizational identification, a sub-variable of OC. Their results showed that employees perform selfless behaviors for an organization only when they develop a sense of belonging. Baruch et al. [67] studied prosocial behavior, a higher OCB construct. They found that OC mediates the relationship between the need for achievement and prosocial behavior, identifying the precedence of OC over employees' performance of extra-role behaviors. Further, the need for achievement directly influences prosocial behavior and job performance. This research shows that employees’ motivation contributes to OC, and OC influences SO OCB. Therefore, we propose H6 as follows.

H6 OC positively relates to SO OCB.

### 3.3 OC as a mediator between ESG activity recognition and SO OCB

Wu and Liu's research [52] examined whether employees' perception of organizational support, a social aspect of ESG, influenced OC and, subsequently, SO 10.13039/100012782OCB. They found that OC fully mediates the relationship between perceived organizational support and SO 10.13039/100012782OCB. Furthermore, organizational identification fully mediates the relationship between perceived CSR and SO OCB [4]. Therefore, we do not perceive that ESG activity recognition directly influences SO OCB. However, in the analysis section, we tested the mediating effect of self-efficacy and empowerment between ESG activity recognition and SO OCB.

## 4 Methodology

A questionnaire was developed based on a thorough review of the related literature. All items utilized a 5-point Likert scale. This research defines ESG activity recognition as employees' recognition of a firm's ESG activities based on transparent governance. Three ESG activity recognition items were adapted from Jin and Kim [3] for the questionnaire. This study defines self-efficacy as employees' feelings of confidence and motivation to handle job-related tasks and challenges. We adopted four self-efficacy scales from Bandura, Freeman, and Lightsey's [39]. Empowerment is defined as employees' use of autonomy for decision-making in performing their duties and tasks. Five questions on empowerment were adapted from Spreitzer [62] for the questionnaire. Furthermore, we define OC as employees' level of identification and involvement with an organization. It was measured with four items from Mowday [68]. Finally, SO OCB utilized two items from Bettencourt and Brown [69]. Based on Organ's [70] research, we defined it as service employees' spontaneous gestures that help sustain a constructive interpersonal climate.

Two managerial-level call center professionals and two academic experts in human resource management reviewed the questionnaire for content validity. Minor revisions were made based on their suggestions. The final items used for the analysis are listed in Table 1. After the revision process, a pilot test was conducted between June and August 2024, with the main data collected over two days in December 2024 using an online survey created with Google Forms. Potential survey participants were contacted using a snowball method (Chonnam National University IRB No. 1040198-231005-HR-141-02). A researcher involved in the project used to be a high-ranking manager at a prominent call center. Thus, this researcher's personal network was used to contact and collect data from customer representatives in several major insurance companies. The study's purpose was fully disclosed to participants before the survey questions were asked, along with explanations of expected minimal risks and benefits, usage and security of personally identifiable information, voluntary participation and removal, contacts for more information, and compensation for possible harm of the study. After reading the information, participants clicked the ‘agree’ button to provide informed consent and begin the survey. Due to the nature of the data collection using an online survey, our survey was approved by the IRB of not collecting actual informed consents from the participants. Overall, 230 call center workers in South Korea responded, and 204 surveys were finally analyzed after removing incomplete responses. Hoogland and Boomsma [71] determined that structural equation analysis is recommended for sample size bigger than 200. Thus, this study's sample size meets this stipulation.

**Table 1:** Measurement items.

Variables	Item ID	Adapted Survey Items	References
ESG Activity Recognition	ESG1	My company adopts the ethical regulations of the employees.	[3] Jin and Kim (2022)
	ESG2	My company discloses information and issues gravely affecting decision-making.	
	ESG3	My company performs continuous disclosures (publishing sustainability management reports) externally.	
Self-Efficacy	SE1	I always manage to solve difficult problems if I try hard enough.	[39] Bandura, Freeman, and Lightsey (1999)
	SE2	It is easy for me to stick to my aims and accomplish my goals.	
	SE3	I am confident that I could deal efficiently with unexpected events.	
	SE4	If I am in trouble, I can usually think of a solution.	
Empowerment	EM1	I have significant autonomy in determining how I do my job.	[62] Spreitzer (1995)
	EM2	I can decide on my own how to go about doing my work.	
	EM3	I have considerable opportunities for independence and freedom in how I do my job.	
	EM 4	I have a significant influence on what happens in my department.	
	EM5	I have a great deal of control over what happens in my department.	
OC	OC1	I am proud to tell others that I am part of this organization.	[68] Mowday et al. (1979)
	OC2	I would be very happy to spend the rest of my career with this organization.	
	OC3	I am willing to put in a great deal of effort beyond that normally expected in order to help this organization be successful.	
	OC4	I find that my values and the organization's values are very similar.	
SO OCB	OCB1	I help customers with problems beyond what is expected or required.	[69] Bettencourt and Brown (1997)
	OCB2	I often go above and beyond the call of duty when serving customers	

Following the two-step approach suggested by Anderson and Gerbing [72], this study used structural equation modeling (SEM) with a maximum likelihood (ML) method to ensure the relationships among constructs. Thus, this study conducted a confirmatory factor analysis (CFA) to measure the validity of the hypothesized latent constructs. Following this, SEM was performed to determine whether the proposed model and relationships were valid.

This study reports several criteria used in STATA 14.0 to assess the goodness of fit, such as chi-square statistics, the Root Mean Square of Error Approximation (RMSEA), the Comparative Fit Index (CFI), the Tucker-Lewis Index (TLI), and the Standardized Root Mean Squared Residual (SRMR).

## 5 Analysis and results

### 5.1 Descriptive statistics

Table 2 describes the study sample [73] profile. All respondents were female. Only 20.10 % were married, and 60.78 % were between 40 and 49 years old. Most participants were customer service representatives with more than 15 years of work experience.

**Table 2:** Respondent descriptions.

Classification		Frequency	Percentage (%)
Gender	Male	0	
	Female	204	100
	Total	204	100
Marital Status	Married	41	20.10
	Single	163	76.90
	Total	204	100
Age	20–29 years	6	2.94
	30–39 years	48	23.53
	40–49 years	164	60.78
	50 years and older	26	12.74
	Total	204	100
Education	High school	40	19.61
	Associate degree	49	24.02
	Bachelor's degree	113	55.39
	Graduate degree	1	0.98
	Total	204	100
Employment Duration	Less than 1 year	7	3.43
	1–5 years	20	9.80
	6–10 years	41	20.10
	11–15 years	49	24.02
	More than 15 years	87	42.65
	Total	204	100
Position	Customer representative	175	85.78
	Management	29	14.22
	Total	204	100

n = 204.

### 5.2 Reliability and validity tests

First, this study tested for reliability. The coefficient alpha is a highly credible measurement of a multi-item scale's reliability. All estimated alpha coefficients were well above the recommended cut-off of 0.70 [74], indicating adequate internal consistency among construct items. Furthermore, all standardized factor loadings exceeded 0.861, ensuring convergent validity [72]. In addition, the average variance extracted (AVE) of all constructs met the minimum criteria of 0.50 [75].

Meanwhile, CFA's goodness-of-fit statistics, which were undertaken by STATA 14.0, presented that the model reasonably fit the data: χ^2^/df = 2.02 (criterion≤3.0), RMSEA = 0.071 (criterion≤0.08), CFI = 0.96 (criterion ≥0.9), TLI = 0.94 (criterion ≥0.9), and SRMR = 0.05(criterion≤0.08) ( [[76], [77], [78], [79]].

Measurement details are shown in Table 3 below.

**Table 3:** The results of the reliability and convergent validity tests.

**Variables**	**Items**	**Standardized Factor Loading**	**CR**	**AVE**	**Cronbach's α**	**Μ(SD)**
ESG Activity Recognition	ESG8	0.860	0.861	0.675	0.857	2.520 (0.91)
	ESG9	0.809				
	ESG10	0.792				
Self-Efficacy	SE1	0.821	0.898	0.688	0.925	2.360 (0.76)
	SE2	0.817				
	SE3	0.861				
	SE4	0.878				
Empowerment	EM1	0.784	0.877	0.588	0.887	2.930 (0.95)
	EM2	0.793				
	EM3	0.869				
	EM5	0.703				
	EM6	0.647				
OC	OC7	0.890	0.914	0.726	0.899	2.471 (0.91)
	OC9	0.829				
	OC10	0.855				
	OC11	0.833				
SO OCB	PS2	0.746	0.878	0.783	0.878	1.988 (0.71)
	PS3	0.711				

Measurement model fit: χ^2^(df) = 240.77, χ^2^/d f = 2.02, RMSEA = 0.07, CFI = 0.96, TLI = 0.94, SRMR = 0.05.

This study also tested discriminant validity by comparing the AVEs’ squared correlation between constructs [80] as shown in Table 4. The AVEs were higher than the squared correlations, implying discriminant validity. Hence, ESG activity recognition, self-efficacy, empowerment, OC, and SO OCB were determined as five distinguishable constructs.

**Table 4:** Discriminant validity.

	1	2	3	4	5	AVE
1 ESG	1	0.084^b^	0.201	0.433	0.239	0.675
2 Self-Efficacy	0.539***^a^	1	0.308	0.462	0.460	0.688
3 Empowerment	0.448***	0.555***	1	0.404	0.135	0.588
4 OC	0.658***	0.680***	0.636***	1	0.379	0.726
5 SO OCB	0.489**	0.678***	0.368***	0.616***	1	0.783

a ***p < .001.

b The underlined higher-half matrix implies the square root of the correlation.

### 5.3 Structural model results and hypotheses testing

**Fig. 1:**
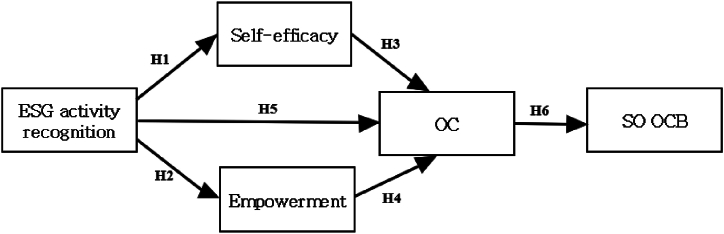
Proposed research mode.

Table 5 summarizes the results of testing the structural model, as depicted in Fig. 1. The diverse goodness-of-fit indices are as follows: χ^2^/df = 3.04 (criterion≤3.0), RMSEA = 0.08 (criterion≤0.08), CFI = 0.94 (criterion ≥0.9), TLI = 0.92 (criterion ≥0.9), and SRMR = 0.092 (criterion≤0.08). All indices indicated a modest fit between the hypothesized model and the data except SRMS. Although SRMS was slightly over the cut-off of 0.08, it appears acceptable. As Gefen et al. [81] mentioned, satisfying all goodness-of-fit indices within the SEM analyses can be difficult.

**Table 5:** Structural parameter estimates.

Classification	Hypothesized Path	Standard Path Coefficient	z-value	Status of Adoption
H1	ESG - > Self-efficacy	0.561	9.48	Adopted
H2	ESG - > Empowerment	0.472	6.88	Adopted
H3	Self-efficacy - > OC	0.356	5.19	Adopted
H4	Empowerment - > OC	0.300	4.52	Adopted
H5	ESG - > OC	0.354	5.23	Adopted
H6	OC - > SO OCB	0.626	12.68	Adopted

1. ***: p<.001, **: p < .01, *p < .05.

2. Structured model fit: χ^2^(df) = 374.372, χ^2^/d f = 3.04, RMSEA = 0.08, CFI = 0.94, TLI = 0.92, SRMR = 0.092.

All hypothesized paths were adopted, as shown in Table 5. The relationships between ESG activity recognition and self-efficacy (Hypothesis 1) and ESG activity recognition and empowerment (Hypothesis 2) were supported by the corresponding estimates of 0.56 (z = 9.48, p < 0.001) and 0.47 (z = 6.88, p < 0.001), respectively. The standard path coefficients of Hypotheses 3 and 4 were 0.036 (z = 5.19, p < 0.001) and 0.30 (z = 4.52, p < 0.001), respectively, suggesting that self-efficacy and empowerment are predictors of OC for call center employees. 10.13039/100014337Furthermore, the direct linkage between ESG activity recognition and OC (Hypothesis 5) was supported by the corresponding estimate of 0.35 (z = 5.23, p < 0.001). In addition, the hypothesized relationship between OC and SO OCB (Hypothesis 6) was adopted based on the corresponding estimate of 0.626 (z = 12.68, p < 0.001).

Lastly, this study further examined the mediating roles of empowerment and self-efficacy on the relationship between ESG activity recognition and OC. Within our model, Hypothesis 1 (*β*_11_) and Hypothesis 3 (*β*_31_) were significant. Hypothesis 5, which suggests the direct effect of ESG activity recognition on OC, was also significant. Under this condition, the indirect effect of ESG activity recognition on OC through self-efficacy was 0.20, resulting from *β*_11_ X *β*_31,_ which was less than the direct effect (0.35). In addition, Hypothesis 2 (*β*_21_) and Hypothesis 4 (*β*_41_) were significant. The indirect effect of ESG activity recognition on OC through empowerment was 0.14, resulting from *β*_21_ X *β*_41_, which was less than the direct effect (0.35). These results testified to the partial mediating roles of self-efficacy and empowerment.

## 6 Discussion

ESG management drives organizational cultural change [28], helping to alleviate pressures employees experience in the workplace [82]. This study examined the role of ESG activities in increasing employee motivation, OC, and, subsequently, SO OCB. As the results show, ESG activity recognition positively influenced the self-efficacy (H1), empowerment (H2), and OC (H5) of employees in the insurance industry experiencing emotional labor. Employees regulate enforced expectations better when they are characterized by self-efficacy and empowerment [[9], [10], [11]]. The support for Hypotheses 1 and 2 suggests that some emotional labor and cognitive dissonance can be reduced when employees positively perceive their organization's culture as ESG-driven. Cognitive dissonance is highly related to the level of emotional labor, and those experiencing cognitive dissonance can actively attempt to address this situation by adopting different attitudes or behaviors [83]. To take a corporate example, Ansarada, an Australian tech firm, incorporated ESG policies, procedures, and systems. By aligning corporate ESG governance with employees' purposes, Andarada, identified as a great place to work, reported increased employee engagement and positive business growth [84].

Self-efficacy and empowerment directly influence OC (H3, H4) and partially mediate the relationship between ESG activity recognition and OC (H5). OC is a highly valued construct in human resources research due to its impact on positive outcomes for employees and organizations. The results of Hypothesis 5 show that recognizing ESG activities alone can significantly enhance OC. Most ESG disclosure focuses on informing external stakeholders about a company's ESG activities. However, our results suggest that employees' recognition of ESG management is also vital. This is academically important as it reveals that an ESG-driven organizational culture promotes service employees' commitment to the organization. OC is a highly researched construct, but it has rarely been studied in connection with ESG. The results of Hypotheses 3, 4, and 5 suggest that educating companies' internal customers about ESG management activities benefits both the organization and internal customers through increased OC. Therefore, HR managers should consider how to enhance employees' recognition of ESG practices to improve OC and obtain its related benefits. Roy et al. [2] point out that ethical and authentic leadership, codes of ethics, and ethics programs and training are predictors of an ethical culture. That is, a top-down approach to adopting ESG management should be supported by an organization's governance structure and ethical training programs for employees.

Ethical leadership is demonstrated through trustworthiness, fairness, concern for others, and ethical intentions. As demonstrated in ESG management, ethical leadership and culture can affect employees' ethical behavior [85]. The final determinant of SO OCB is service employees’ desirable behavior, especially regarding contact employees. The adoption of Hypothesis 6 shows that OC plays a positive role in promoting SO OCB. The results imply that SO OCB can be cultivated within an ethical culture brought about by ESG adoption.

## 7 Conclusion

This research examined the role of ESG activity recognition regarding motivational constructs (self-efficacy and empowerment), attitudinal constructs (OC), and behavioral constructs (SO OCB) among call center employees in South Korea. Academically, research on ESG has focused on increasing organizations' financial performance. The current research contributes to academia by examining ESG's role in 1) addressing emotional labor among workers; 2) boosting OC, an important predictor of employee and organizational performance; 3) contributing to corporate values by improving non-financial performance; and 4) enhancing SO OCB, which can improve external stakeholders' value. Furthermore, this study examined the significance of ESG management in transforming the organizational climate via employees' ESG activity recognition.

Based on the results, the following research identified several practical implications regarding the characteristics of the insurance industry. Employees in insurance call centers engage in emotional labor, adversely affecting their motivation and work-life quality. ESG activity recognition can result in positive motivational changes for workers experiencing emotional labor, increasing their OC. Therefore, this study's findings can serve as an essential basis for helping organizations in the service industry manage employees' emotional labor by creating a better organizational culture through ESG management. Additionally, OC is a good predictor of multiple performance measures, including SO OCB. However, other positive outcomes may result from OC based on dependent variables untested in this research. Thus, future research is required to test OC's dependent variables with other variables to give generalizability to the current research model. Also, additional research should examine what methods are more effective for increasing the recognition of ESG activities and what specific dimensions of OC can help improve SO OCB.

This research has several limitations. First, all survey participants are female full-time workers, reflecting the unique job characteristics of call center work in South Korea. Thus, future studies should enhance the generalizability of the current research's findings. First, the model should be tested with a population outside South Korea to generalize the results. Secondly, this research collects individual employees' ESG activity perceptions to capture their shared corporate culture as a community. This is a persistent limitation of organizational culture-related studies [86]. Thirdly, this study uses ESG activity recognition as a proxy for an ESG-driven corporate climate. While there are a limited number of ESG culture-related studies including various aspects of corporate climate measurements, further studies can be developed to understand the ESG climate more comprehensively. Finally, the study only tests ESG activity recognition regarding governance activities. While corporate climate is typically studied in the context of corporate governance, testing the role of environmental and societal activity recognition would be meaningful. Therefore, future studies can be conducted to determine employees' recognition of these factors.

This study's significant novel findings are as follows. One, the study establishes the relationship between corporate ESG management and OC as well as SO OCB, highlighting the non-financial benefits of ESG adoption. This new finding contributes to the scarcely studied area of micro-ESG research regarding, where the influence of ESG is assessed at the micro level. The other, our finding suggests the role of the new corporate climate brought by ESG management in enhancing corporations' non-financial performances.

Adopting ESG management within an organization requires a cultural shift and employee acceptance. Jobs can be designed to influence employees' perceptions of their social impact and worth and, consequently, their job performance. Employees who identify with organizational goals and realize their social impacts and value may feel empowered to perform tasks. Motivated employees will also be committed to the organization's goals and more willing to engage in OCB.

## Ethics statement

The study was approved by Chonnam National University IRB No: 1040198-231005-HR-141-02 on December 4th^,^ 2023.

## Ethics and consent

The wavier of informed consent is approved by the above listed IRB approval.

## Data availability statement

Data is available at Mendeley Data (https://doi: 10.17632/hvwjmxjvyp.1).

## CRediT authorship contribution statement

**Serin Choi:** Writing – review & editing, Writing – original draft, Software, Methodology, Formal analysis. **Kyeong-Sook Jeong:** Validation, Investigation, Data curation. **So Ra Park:** Writing – review & editing, Writing – original draft, Supervision, Project administration, Investigation.

## Declaration of competing interest

The authors declare that they have no known competing financial interests or personal relationships that could have appeared to influence the work reported in this paper.
